# *Clostridium difficile* – From Colonization to Infection

**DOI:** 10.3389/fmicb.2018.00646

**Published:** 2018-04-10

**Authors:** Holger Schäffler, Anne Breitrück

**Affiliations:** ^1^Division of Gastroenterology, Department of Medicine II, University of Rostock, Rostock, Germany; ^2^Extracorporeal Immunomodulation Unit, Fraunhofer Institute for Cell Therapy and Immunology, Rostock, Germany; ^3^Institute of Medical Microbiology, Virology and Hygiene, University of Rostock, Rostock, Germany

**Keywords:** *Clostridium difficile*, microbiota, *Clostridium difficile* infection, CDI, asymptomatic colonization

## Abstract

*Clostridium difficile* is the most frequent cause of nosocomial antibiotic-associated diarrhea. The incidence of *C. difficile* infection (CDI) has been rising worldwide with subsequent increases in morbidity, mortality, and health care costs. Asymptomatic colonization with *C. difficile* is common and a high prevalence has been found in specific cohorts, e.g., hospitalized patients, adults in nursing homes and in infants. However, the risk of infection with *C. difficile* differs significantly between these cohorts. While CDI is a clear indication for therapy, colonization with *C. difficile* is not believed to be a direct precursor for CDI and therefore does not require treatment. Antibiotic therapy causes alterations of the intestinal microbial composition, enabling *C. difficile* colonization and consecutive toxin production leading to disruption of the colonic epithelial cells. Clinical symptoms of CDI range from mild diarrhea to potentially life-threatening conditions like pseudomembranous colitis or toxic megacolon. While antibiotics are still the treatment of choice for CDI, new therapies have emerged in recent years such as antibodies against *C. difficile* toxin B and fecal microbial transfer (FMT). This specific therapy for CDI underscores the role of the indigenous bacterial composition in the prevention of the disease in healthy individuals and its role in the pathogenesis after alteration by antibiotic treatment. In addition to the pathogenesis of CDI, this review focuses on the colonization of *C. difficile* in the human gut and factors promoting CDI.

## Introduction

*Clostridium difficile* was first described as part of the intestinal bacterial composition in newborns in 1935 (Hall and O′Toole, 1935). In the 1970s, *C. difficile* was identified as the causative agent for pseudomembranous colitis following antibiotic therapy. The pathogenic potential of this strain was proven to fulfill the Koch’s postulates (Bartlett et al., 1977) underscoring its role in the development of CDI. Recently, a dramatic increase in the incidence as well as in the mortality of CDI could be observed worldwide (Ananthakrishnan, 2011; Lo Vecchio and Zacur, 2012; Tattevin et al., 2013).

The clinical spectrum of *C. difficile* ranges from asymptomatic colonization, mild and self-limiting disease to a severe, life-threatening pseudomembranous colitis, toxic megacolon, sepsis and death (Gerding et al., 1995; Rupnik et al., 2009). CDI is defined when there is the presence of symptomatic diarrhea defined by three or more unformed stools per 24 h and at least one of the following criteria: a positive laboratory assay for *C. difficile* toxin A and/or B or toxin-producing *C. difficile* organism in a stool sample or pseudomembranous colitis or colonic histopathology characteristics of CDI revealed by endoscopy (Kuijper et al., 2006; Cohen et al., 2010). CDI is associated with an increased abundance of toxin-producing *C. difficile* strains, leading to high toxin concentrations within the colon resulting in inflammation and damage of the colonocytes (Ishida et al., 2004; Meyer et al., 2007; Carroll and Bartlett, 2011). Usually, the indigenous microbial communities provide a colonization resistance to *C. difficile*, which could also be proven in animal models (Wilson et al., 1981). However, a disruption of this microbial system can promote the development of CDI (Rea et al., 2012; Buffie et al., 2015).

While the clinical presentation of CDI is distinctive, *C. difficile* colonization without any symptoms, defined as asymptomatic colonization is common, especially in neonates (Ozaki et al., 2004; Jangi and Lamont, 2010). This review focuses on the role of *C. difficile* in asymptomatic colonization and CDI to better understand which factors might contribute to the progression and also prevention of the disease.

## Microbiology

*C. difficile* is a Gram-positive, anaerobic, spore-forming and toxin-producing bacillus, belonging to cluster XI the *Clostridium* genus and can be isolated from water, vegetables, hospital environment, and the intestines of humans and domesticated animals (Weese, 2010).

Different virulence factors are associated with the development of CDI. The most important virulence factor is the release of multiple toxins, namely large glycosylating exotoxins A (TcdA) and B (TcdB). These toxins lead to the characteristic clinical symptoms by binding to *C. difficile* toxin receptors on intestinal epithelial cells (Kelly and LaMont, 1998; Voth and Ballard, 2005; Pruitt and Lacy, 2012; Shen, 2012). Another toxin can be found in some *C. difficile* strains, especially the PCR ribotype 027, named binary toxin or *C. difficile* transferase, which is associated with a higher mortality rate in patients (Gerding et al., 2014). This ribotype 027 carries a deletion in tcdC, which is discussed to play a major role in its increased production of toxins (Warny et al., 2005; Curry et al., 2007; Dupuy et al., 2008). There are *C. difficile* strains which can synthesize *C. difficile* transferase in the absence of TcdA and TcdB (McFarland et al., 2007a). These toxins, which are encoded on the pathogenicity locus, are multi-domain toxins with glycosyltransferase activities, which transfer glycosyl residues to small Ras homologous GTPases and consecutively lead to a loss of the intestinal membrane integrity and to cell death (Schirmer and Aktories, 2004; Moore et al., 2013). As a consequence, inflammation of the colon occurs with massive fluid loss into the large intestine, clinically presenting as acute diarrhea (Voth and Ballard, 2005). While initially TcdA was suggested to play a more prominent role in the development of CDI compared to TcdB, this view was challenged by different other studies (Lyerly et al., 1985; Komatsu et al., 2003; Drudy et al., 2007; Lyras et al., 2009; Steele et al., 2013). Colonization with *C. difficile* occurs by oral ingestion of spores from infected individuals or the environment (Jump et al., 2007; Gerding et al., 2008; Sarker and Paredes-Sabja, 2012). While *C. difficile* is an anaerobic organism, *C. difficile* spores can survive in aerobic environments for months or years (Rupnik et al., 2009). *C. difficile* spores are resistant to the gastric acid and can germinate into vegetative cells in the anaerobic conditions of the colon (Gil et al., 2017).

## Epidemiology of CDI

The worldwide incidence of CDI has been rising steadily since 2000, however, susceptibility to treatment decreased (McDonald et al., 2006; Vardakas et al., 2012; Bagdasarian et al., 2015). *C. difficile* was first reported to cause severe antibiotic-associated diarrhea and pseudomembranous colitis in the 1970s and has become the most common healthcare-associated infection, leading to about 500,000 cases and 29,000 deaths annually in the United States (Bartlett et al., 1978; Bartlett, 2006; Lessa et al., 2015). Overall, the epidemiology data of CDI in Europe are more variable due to different reporting systems within the European Union. However, by extrapolation of the data from the United Kingdom to Europe, they result in a total number of 172,000 CDI cases annually within the European Union (Barbut et al., 2013). The epidemic spread of hypervirulent *C. difficile* strains, e.g., PCR ribotype 027 leads to larger nosocomial outbreaks, which are associated with increased morbidity and mortality (Reichardt et al., 2007; Bacci et al., 2011). The economic impact of CDI is enormous, leading to additional medical costs of over one billion dollar per year in the United States and three billion euro per year within the European Union (Kuijper et al., 2006; Dubberke and Olsen, 2012). Especially hospitalized patients and adults in long-term care facilities are at a higher risk of developing CDI (Simor et al., 2002; Bauer et al., 2011; Kim et al., 2011). Additionally, in infants an increase of CDI was observed in the last decade (Zilberberg et al., 2008; Nylund et al., 2011; Khanna et al., 2013; McFarland et al., 2016). This is especially interesting since – as mentioned above – *C. difficile* is highly prevalent in infants, however, they usually do not show clinical signs of CDI. Nevertheless, the data regarding CDI in pediatric patients are limited.

## Asymptomatic Colonization

While many studies have focused on the pathogenesis and the development of CDI, the role of asymptomatic *C. difficile* colonization and its progression to CDI is still not completely understood. While in the past literature the definition of asymptomatic *C. difficile* colonization is not uniform, Furuya-Kanamori et al. (2015) were proposing one as followed: either detectable concentrations of *C. difficile* or its toxin and the absence of diarrhea without colonoscopic or histopathologic findings consistent with pseudomembranous colitis.

The prevalence of asymptomatic *C. difficile* colonization in adults varies in different population groups. In healthy adults, several studies have shown that 0–17.5% were colonized by *C. difficile* strains without clinical signs of CDI (Nakamura et al., 1981; Viscidi et al., 1981; Kobayashi, 1983; Aronsson et al., 1985; Fekety and Shah, 1993; Ozaki et al., 2004; Terveer et al., 2017). The colonization rate of toxigenic strains ranges from 1 to 5% in the surveyed group. While the prevalence of asymptomatic *C. difficile* colonization is relatively low in healthy adults, it can rise dramatically in individuals having contact with the health system. Elderly in long-term care facilities or nursing homes have an increased rate of colonization range from 0 to 51% (Campbell et al., 1988; Riggs et al., 2007; Arvand et al., 2012). A high prevalence of asymptomatic *C. difficile* can also be found in patients or health-care workers (McFarland et al., 1989; Samore et al., 1994; Kato et al., 2001; Hell et al., 2012; Guerrero et al., 2013; Leekha et al., 2013). Additionally, patients in rehabilitation centers have an increased rate of asymptomatic *C. difficile* colonization (Marciniak et al., 2006; Stevens et al., 2011). Furthermore, a high percentage of asymptomatic *C. difficile* colonization can be found in adult patients with underlying diseases, e.g., cystic fibrosis (Welkon et al., 1985; Peach et al., 1986; Bauer et al., 2014).

Risk factors for the development of the last asymptomatic *C. difficile* colonization are hospitalization within 12 months, use of corticosteroids, a previous history of CDI and antibodies against toxin B (Kong et al., 2015).

In contrast to adults, a high prevalence of *C. difficile* colonization without clinical signs of CDI can be observed in infants and neonates (Thompson et al., 1983; Rousseau et al., 2012). Especially in the first 4 weeks of life, *C. difficile* colonization increases from 0% to an average of 37%, followed by a recovery, dropping to an average of 10% during the first year of life (Bolton et al., 1984; Jangi and Lamont, 2010). Colonization rates comparable to the rates in healthy adults were observed in infants by the age of 2 (Hafiz and Oakley, 1976). Analysis of ribotypes revealed that pediatric patients harbor several toxigenic strains that circulate in adult patients (Schwartz et al., 2014; van Dorp et al., 2017).

Although – according to the definition – asymptomatic colonized individuals show no clinical signs of CDI, they can act as a reservoir of *C. difficile* and also may serve as potential disease carriers and might therefore transmit *C. difficile* to others (McFarland et al., 1989; Riggs et al., 2007; Curry et al., 2013; Eyre et al., 2013). Furthermore, asymptomatic colonization with *C. difficile* is a crucial factor in the progression to CDI, as carriers of toxigenic strains are at a higher risk for the development of an infection compared to non-colonized patients (Zacharioudakis et al., 2015).

In contrast to this, asymptomatic colonization could also induce the production of antibodies, which in turn protect their host against CDI with a humoral immune response (Shim et al., 1998; Kyne et al., 2000, 2001). It was also proposed that asymptomatic *C. difficile* colonization might lead to the integration of *C. difficile* into the indigenous intestinal microbiota, serving as a protective factor for the development of CDI (Vincent et al., 2016). This theory could also be proven in hamsters, where colonization with a non-toxigenic *C. difficile* strain before the application of a toxigenic *C. difficile* strain was able to prevent the development of CDI (Sambol et al., 2002; Merrigan et al., 2003, 2009). However, further prospective studies to better understand the mechanisms how individuals develop asymptomatic *C. difficile* colonization and if this may act as risk or protective factor in the progression for an infection are needed.

## From Colonization to Infection

**FIGURE 1:**
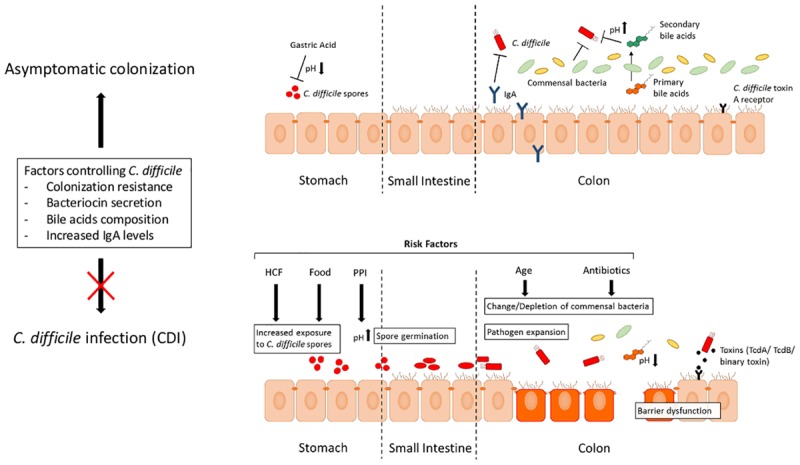
Processes leading from asymptomatic *C. difficile* colonization to CDI. Different factors can prevent an asymptomatic individual from the development of CDI. Gastric acid production within the stomach prevents further spreading of the spores. A healthy indigenous intestinal microbial composition serves as a colonization resistance, can produce bacteriocins limiting *C. difficile* expansion and compete with nutritional contents. Additionally, a change in the bile acid composition can also have effects on the expansion of *C. difficile*. Increased exposure to *C. difficile*, e.g., contact with HCF or via oral ingestion with food, predisposes an individual at risk for asymptomatic colonization. In case of underlying risk factors, an asymptomatic colonization can progress to CDI. After application of antibiotics, a depletion of the commensal bacterial composition can occur leading to a reduced colonization resistance favoring the development of CDI. Other risk factors are increased age, comorbidities and the application of drugs which reduce the gastric acid, e.g., proton pump inhibitors (PPIs). Abbreviations: CDI, *C. difficile* infection; HCF, health care facilities; IgA, Immunoglobulin A; PPI, proton pump inhibitor; TcdA, *C. difficile* toxin A; TcdB, *C. difficile* toxin B.

A schematic overview which factors lead to the development of CDI is given in **Figure 1**.

### Host Factors

#### The Indigenous Microbiota

The intestinal microbiota is a complex ecosystem consisting of over a thousand bacterial species reaching its highest concentration in the colon (Ley et al., 2006; Sekirov et al., 2010; Blaser, 2014). In adults, a healthy intestinal microbiota is dominated by the phyla Bacteroidetes and Firmicutes and shows a high diversity and richness (Rajilić-Stojanović et al., 2009). These commensal bacteria are essential for the host metabolism, nutrition function, maturation of the immune system and protection against pathogens. During human lifetime, different factors, such as the mode of delivery, diet, geography, antibiotic use and the development of gastrointestinal diseases can influence the composition of the intestinal microbiota (Schultsz et al., 1999; Huurre et al., 2008; De Filippo et al., 2010; Dominguez-Bello et al., 2010; Jakobsson et al., 2010; Manges et al., 2010; Dethlefsen and Relman, 2011; Muegge et al., 2011; Wu et al., 2011; Yatsunenko et al., 2012; Zupancic et al., 2012; Matamoros et al., 2013; Ringel and Maharshak, 2013). A disruption of this ecosystem, a so-called intestinal dysbiosis can have a significant influence on the structure and the function of the resident microbiota. Changes of the indigenous intestinal microbial composition result in a breakdown of the colonization resistance, which favors *C. difficile* germination, growth and spreading within the intestine (Antonopoulos et al., 2009; Robinson and Young, 2010).

It is well known that the susceptibility of CDI is strongly associated with a previous exposure to antibiotics. The application of antibiotics, especially broad-spectrum antibiotics, can have profound and long-lasting consequences on the host by altering the intestinal bacterial composition and the metabolome (Dethlefsen et al., 2008; Antonopoulos et al., 2009; Peterfreund et al., 2012; Theriot et al., 2014). The first description of CDI in the setting of antibiotic therapy was in 1974 in a patient after the application of clindamycin (Tedesco et al., 1974). Other antibiotics, especially cephalosporins, penicillin and fluoroquinolones have also been reported to be associated with the development of CDI (Bartlett, 2010; Manges et al., 2010). Moreover, it has been demonstrated that cumulative exposure to any kind of antibiotics increases the risk of developing CDI (Stevens et al., 2011). The impact of antibiotic administration on the microbial composition in healthy adults has been studied extensively. Sullivan et al. (2001) summarized the effects of different antimicrobial agents on the bacterial diversity and single bacterial phylae, families and classes. The effects of antibiotics on the intestinal microbial composition can also be investigated in murine models, allowing the examination of factors which lead to the resistance of *C. difficile* colonization and subsequent development of CDI (Chen et al., 2008; Reeves et al., 2011, 2012; Buffie et al., 2012; Lawley et al., 2012; Winston et al., 2016). Different studies described the intestinal microbial composition in patients with CDI and observed similar results regarding a reduced diversity and also reduced species richness. In contrast to healthy controls, patients with CDI showed an increase in Firmicutes and Proteobacteria phyla and a decrease in Bacteroidetes phylum. Furthermore, *Ruminococcacea*, *Lachnospiraceae*, *Bacteriodaceae*, Clostrida cluster IV and XIVa are decreased while an increased abundance of Enterococacea could be observed (Chang et al., 2008; Antharam et al., 2013; Hamilton et al., 2013). Additionally, in a study by Vincent et al. (2013, 2016), co-colonization with potentially protective bacterial taxa, e.g., Clostridiales Family XI Incertae Sedis, Clostridium or Eubacterium may protect the development of CDI from asymptomatic *C. difficile* colonization. In a recent study by Pakpour et al. (2017), *Veillonella dispar* was found as a candidate organism which might be protective for the recurrence of *C. difficile*.

#### Bile Acids

Germination of *C. difficile* spores is supported by changes in the composition of bile acids (Jump et al., 2007; Howerton et al., 2011). A reduced number of bacteria, producing hydrolase enzymes, results in a reduction of secondary bile acids, which normally inhibit vegetative cell growth and a simultaneous increase of primary bile acids like cholate or taurocholic acid stimulates spore germination (Sorg and Sonenshein, 2008). While cholate and glycine can promote *C. difficile* spore formation, chenodeoxycholate was found to act as an inhibitor of spore formation (Wilson, 1983; Sorg and Sonenshein, 2008). A depletion of commensals can also result in an oversupply of available nutrients, e.g., monosaccharides, which can further be utilized by *C. difficile* (Wilson and Perini, 1988; Begley et al., 2006). In a murine model, administration of antibiotics led to a shift of the bile acid pool and therefore increased *C. difficile* spore germination (Giel et al., 2010; Antunes and Finlay, 2011).

#### Bacteriocins

Bacteriocins are ribosomally synthesized antimicrobial peptides with narrow or broad spectrum activity against other bacterial species (Bacon et al., 1988; Cotter et al., 2005). The secretion of bacteriocins from *Bacillus*, *Lactococcus*, and *Enterococcus* strains, whose antimicrobial function against *C. difficile* has been proven *in vitro*, is decreased in CDI (Bartoloni et al., 2004; Rea et al., 2007; Trzasko et al., 2012).

#### *C. Difficile* Toxin A Receptor

The susceptibility of the host to CDI is also associated with the presence or absence of *C. difficile* toxin A receptor on the surface of intestinal epithelial cells. This fact was reinforced by the study of Eglow et al. (1992) which compared the effect of toxin A in newborn compared to adult rabbit ileum. The absence of pathological effects in the newborn ileum may be due to a complete lack or only a low expression of *C. difficile* toxin A receptor and therefore might prevent neonates from the development a CDI.

#### Immunological Factors

It is well known that the host recognition of *C. difficile* and the subsequent innate and adaptive immune responses have a protective effect against the development of CDI (Cowardin and Petri, 2014; Buonomo and Petri, 2016). Kyne et al. (2000) tested prospectively the immune response of the host via measuring IgG antibodies of TcdA and found that an acquired antibody response to *C. difficile* protected against CDI. However, the antibody response to *C. difficile* did not affect asymptomatic *C. difficile* colonization.

The protective effect of high IgA concentrations, which is a potent toxin A neutralizer has also been shown in breastfeed infants (Rolfe and Song, 1995). The presence of maternal secretory IgA may prevent them from colonization, as this cohort was found to have lower recovery rates compared to formula-fed infants (Viscidi et al., 1981; Larson et al., 1982; Stark and Lee, 1982; Richardson et al., 1983; Wongwanich et al., 2001). However, beneath IgA also other components of breast milk have the potential to bind *C. difficile* toxin A (Rolfe and Song, 1995; Naaber et al., 1996).

### Bacterial Factors

The development of CDI is closely linked to the bacterial virulence factors TcdA and TcdB and the binary toxin, as non-toxigenic *C. difficile* strains are not known to cause CDI (Geric et al., 2006). Colonization with hypervirulent ribotype NAP1 occurred more likely in CDI than in asymptomatic colonized individuals, due to an increased TcdA and TcdB production compared to other toxigenic ribotypes (Warny et al., 2005; Loo et al., 2011; Alasmari et al., 2014).

### Extrinsic Risk Factors

Different studies indicate that – beneath host-mediated and pathogen-related factors – multiple extrinsic risk factors increase the development and also severity of CDI.

#### Antibiotics

As described above, the use of antibiotics is the most-common risk factor in the development of CDI. Antibiotics have dramatic effects on the bacterial ecosystem of the gut, which can last for a long period of time (Dethlefsen et al., 2008; Antonopoulos et al., 2009). Especially fluoroquinolones and particularly cephalosporins and clindamycin are associated with an increased frequency of CDI (Nelson et al., 1994; McCusker et al., 2003; Muto et al., 2005; McFarland et al., 2007b; Kallen et al., 2009).

#### Proton Pump Inhibitors

Another important risk factor for the development of CDI is the use of proton pump inhibitors (PPIs) (Dial et al., 2004; Akhtar and Shaheen, 2007; Deshpande et al., 2012). While the normal gastric acidity provides a protective host defense, an increase of the gastric pH may prevent the gastric content from an elimination of the ingested *C. difficile* spores (Bavishi and Dupont, 2011). However, the role of PPIs in the development of CDI is still controversial, since other studies could not prove an association between the gastric acid suppression and an increased risk for the development of CDI (Novack et al., 2014; Khanafer et al., 2017). Since the use of PPIs is increasing globally, further prospective studies are needed in order to address the possible association with these drugs and the development of asymptomatic *C. difficile* colonization or CDI.

#### Health Care Facilities

In hospitals or long-term care facilities, an increased exposure to *C. difficile* can be found due to high *C. difficile* contamination on surfaces, medical devices and health care personal or infected roommates (McFarland et al., 1989; Chang and Nelson, 2000). Furthermore, a high rate of polypharmacy like antibiotics and underlying co-morbidities such as malignancy or inflammatory bowel disease are closely associated with patients in health care facilities (Morris et al., 1984; Rea et al., 2007). To decrease the *C. difficile* transmission and infection rate in hospitals and long-term care facilities, a screening of new patients could be an option to identify toxigenic strain carriers and isolate them from other patients. These approach was able to significantly decrease the incidence of hospital acquired CDI in a prospective Canadian study cohort (Longtin et al., 2016).

#### Age

*Clostridium difficile* is more common in advanced age, also showing a more severe outcome in this population (Loo et al., 2005; Pépin et al., 2005; Henrich et al., 2009; Miller et al., 2010). There are several possible mechanisms for this phenomenon. First, an inadequate innate or humoral immune response might lead to a higher incidence and also severity of CDI (Kelly, 1996; Mariat et al., 2009; Ogra, 2010). Secondarily, the higher prevalence of CDI in the elderly could also be associated with the change of the intestinal microbial composition, e.g., loss of bacterial diversity during aging, which might promote *C. difficile* colonization (Hopkins et al., 2001; Woodmansey, 2007). Additionally, the presence of chronic disorders and an increase in the infection rate, requiring polypharmacy, including antibiotics, is generally much higher in this age cohort (Garibaldi and Nurse, 1986; Werner and Kuntsche, 2000; Gao et al., 2018).

#### *C. difficile* in Food

While the transmission of *C. difficile* from humans to humans is well-established, *C. difficile* as a foodborne disease still remains a matter of debate. In different studies, *C. difficile* was found in retail meat (Rodriguez-Palacios et al., 2007, 2009). Additionally, *C. difficile* was also detected in water, vegetables, pets and also piglets (Borriello et al., 1983; al Saif and Brazier, 1996; Keel et al., 2007; Songer et al., 2007; Yaeger et al., 2007; Clooten et al., 2008; Pirs et al., 2008; Bakri et al., 2009). Regular exposure to *C. difficile* in the food might lead to asymptomatic *C. difficile* colonization. However, since community-acquired *C. difficile* is relatively uncommon, it is not clear if the ingestion of *C. difficile* via the oral route also leads to consecutive CDI. Further studies will be needed in order to address this issue.

## Treatment of CDI

### Conventional

The mainstay in the treatment of CDI is – beneath the withdrawal of antibiotics fostering CDI – the initiation of an antibiotic therapy, e.g., vancomycin or metronidazole (Debast et al., 2014; Hagel et al., 2015). Recent advances in the therapy of *C. difficile* and the role of antibiotic resistance in CDI are summarized elsewhere (Spigaglia, 2016). However, therapy of recurrent CDI can be challenging with conventional antibiotic therapy (Cohen et al., 2010; Wilcox et al., 2017). Recurrence of CDI is found in 20–30% of the patients with a high mortality rate in this cohort (Dubberke and Olsen, 2012). Fidaxomicin, approved by the United States Food and Drug Administration for CDI treatment, shows reduced recurrence rates in patients with *C. difficile*, however, not in the highly virulent strains B1/NAP1/027 (Louie et al., 2011; Cornely et al., 2012). In a recent study, the use of bezlotoxumab, a human monoclonal antibody against TcdB, was associated with a lower rate of *C. difficile* recurrent infection compared to placebo (Wilcox et al., 2017). The addition of an antibody against TcdA (actoxumab) had no effect on the disease recurrence alone or in combination with bezlotoxumab, which is also underscoring the crucial role of toxin B in the pathogenesis of CDI. A matter of debate for this new approach is the potential combination with fecal microbial transplantation (FMT). Further studies will be needed in order to redefine the treatment algorithm of CDI with bezlotoxumab.

### Microbiota-Targeted Therapy

The intestinal microbial communities of patients with CDI differ from patients with asymptomatic *C. difficile* colonization (Rousseau et al., 2012). In different studies, the administration of single strain probiotics showed only limited success in the treatment of CDI (Pochapin, 2000; Wullt et al., 2003). The role of probiotics in the prevention of CDI is still discussed controversial (Shen et al., 2017; Vernaya et al., 2017). In contrast to this, the probiotic treatment with three strains from Lactobacillus parallel to antibiotic application in hospitalized adults showed a significantly decreased CDI rate from 18.0 to 2.3 cases per 10,000 patients-days during the 10-year observation period (Maziade et al., 2015). The most direct and effective way in changing the patient’s intestinal bacterial composition is via FMT. FMT is highly effective in the treatment of antibiotic-refractory CDI and recently was also shown to be cost effective (Kassam et al., 2013; van Nood et al., 2013; Arbel et al., 2017). FMT involves installation of stool from a healthy donor into a patient, leading to a shift of the intestinal microbial communities. Despite the high effectiveness of FMT in the treatment of recurrent CDI, the long-term effects of this therapeutic approach are still not known and might lead to an increased risk of other diseases. Furthermore, FMT is still a highly diverse biological product with several challenges in the standardization of protocols (Arbel et al., 2017). Another therapeutic approach is the administration of non-toxigenic *C. difficile* strains or a mixture of spore-forming commensals. In two phase II clinical trials testing both treatments, a significant decrease of CDI recurrence was observed (Gerding et al., 2015; Khanna et al., 2016). However, in another study it was observed that non-toxigenic strains had the capacity to change their phenotype to toxigenic *C. difficile* strains (Brouwer et al., 2013). Therefore, non-toxigenic strains can also be a predisposition in the development of CDI and have to be used with caution in the setting of *C. difficile* prevention.

## Conclusion

The incidence of CDI increased dramatically in the last years. While asymptomatic *C. difficile* colonization is common especially in newborns, the progression from asymptomatic colonization to infection is not completely understood and large, prospective studies are lacking. While many studies in adults and infants showed high *C. difficile* colonization rates with toxigenic as well as non-toxigenic strains, the detection of *C. difficile* or its toxins in feces of individuals does not immediately implicate an infection with this pathogen and therefore treatment is only indicated when there are clinical signs of CDI. Although persons with asymptomatic *C. difficile* colonization are potential disease carriers and therefore predispose a risk factor for themselves and other people, based on current information an eradication of *C. difficile* is not indicated. CDI is strongly associated with host-mediated factors, including the indigenous microbiota, bacteriocins, toxin A receptor and immunological factors as well as pathogen-related factors, including TcdA, TcdB, and binary toxin. However, host-mediated factors are discussed to have a more pronounced role in the development of CDI (McFarland et al., 1991; Cheng et al., 1997). Especially a disruption of the indigenous intestinal microbial composition within the host can promote the development of CDI via germination and proliferation of toxigenic *C. difficile* strains. Furthermore, several external factors like age, polypharmacy or underlying medical conditions increase the risk and severity of CDI. Due to the strong association between CDI and antibiotic exposure, therapeutic approaches that target the modulation of the intestinal bacterial composition like FMT are crucial in this clinical setting. Treatment strategies with non-toxigenic *C. difficile* strains are on their way, however, a change of non-toxigenic to toxigenic *C. difficile* strains can occur, making this therapeutic approach challenging.

The role of asymptomatic *C. difficile* colonization in the development of CDI is still a controversial matter of debate. Further studies elucidating the clinical consequences of asymptomatic *C. difficile* colonization are needed to further investigate if the presence of *C. difficile* without any signs of CDI is beneficial or might potentially be harmful. We conclude that mechanisms that enable the progression from asymptomatic *C. difficile* colonization to CDI are closely associated with host-mediated as well as pathogen-related factors and a combination of both might be of outstanding interest in the pathogenesis and also prevention of CDI.

## Author’s Note

Due to a limitation of words no citation of all primary literature was possible, the authors kindly ask to excuse this circumstance.

## Author Contributions

All authors listed have made a substantial, direct and intellectual contribution to the work, and approved it for publication.

## Conflict of Interest Statement

The authors declare that the research was conducted in the absence of any commercial or financial relationships that could be construed as a potential conflict of interest.

## Abbreviations

*C. difficile*: *Clostridium difficile*
CDI: *C. difficile* infection
FMT: fecal microbiota transfer
Ig: immunoglobulin
TcdA: *C. difficile* toxin A
TcdB: *C. difficle* toxin B.
